# Short-Chain Fatty Acids in Heart Failure with Preserved Ejection Fraction: Pathophysiological Roles and Therapeutic Potential

**DOI:** 10.3390/ijms27177736

**Published:** 2026-08-29

**Authors:** Yuzhu Lyu, Jingchen Hou, Ina Ma, Tania Mostoufi, Yuan Jiang, Xiaomei Yang, Wei Dong Gao

**Affiliations:** 1 School of Basic Medical Sciences, Capital Medical University, Beijing 100069, China; lyzhu1786465@gmail.com; 2 Department of Neuroscience, Swarthmore College, Swarthmore, PA 19081, USA; houjingchen098@gmail.com; 3 School of Public Health, The College at Brown University, Providence, RI 02912, USA; ina_ma@brown.edu; 4 Department of Biology, University of Maryland College Park, College Park, MD 20742, USA; taniamostoufi@gmail.com; 5 Meinig School of Biomedical Engineering, Cornell University, Ithaca, NY 14853, USA; yj524@cornell.edu; 6 Translational Research Program, Department of Anesthesiology, Center for Shock, Trauma and Anesthesiology Research, University of Maryland School of Medicine, Baltimore, MD 21201, USA; 7 Department of Anesthesiology and Critical Care Medicine, Johns Hopkins University School of Medicine, Baltimore, MD 21287, USA

**Keywords:** heart failure with preserved ejection fraction, short-chain fatty acid, G-protein-coupled receptor, inflammation, mitochondrial metabolism

## Abstract

Heart failure with preserved ejection fraction (HFpEF) is a clinical syndrome characterized by typical heart failure symptoms, left ventricular ejection fraction (LVEF) ≥50%, elevated natriuretic peptides, and impaired diastolic function. HFpEF constitutes a heterogeneous clinical syndrome with increasing prevalence worldwide and limited therapeutic options. Short-chain fatty acids (SCFAs), generated by gut microbial fermentation of dietary fibers, have recently been implicated as modulators of cardiovascular physiology and pathology. In this narrative review, we synthesize evidence linking SCFAs and SCFA-related signaling to HFpEF pathophysiology, with attention to the distinction between direct HFpEF evidence and findings extrapolated from broader cardiovascular and metabolic models. Although HFpEF-specific data remain limited, available evidence suggests that SCFAs may be relevant to several disease-associated processes, including systemic inflammation, adipokine dysregulation, endothelial dysfunction, myocardial stiffness, and metabolic derangement. We further discuss receptor-mediated and histone deacetylase-dependent mechanisms, consider differences among acetate, propionate, and butyrate, and evaluate the current limitations and therapeutic implications of SCFA-related pathways in HFpEF. At present, SCFA-directed interventions should be viewed as investigational rather than established therapeutic strategies for HFpEF.

## 1. Introduction

Heart failure with preserved ejection fraction (HFpEF) is a clinical syndrome characterized by heart failure (HF) symptoms including dyspnea (shortness of breath), fatigue, and peripheral edema in the presence of a left ventricular ejection fraction (LVEF) ≥50%, elevated levels of natriuretic peptides, and evidence of diastolic dysfunction [1,2]. Age, hypertension, obesity, diabetes, coronary artery disease, and pulmonary or renal diseases are risk factors for HFpEF [3,4]. With the population aging and the increasing prevalence of multiple comorbidities among the 56.2 million patients with HF globally, the incidence of HFpEF continues to rise, affecting >50% of all patients with HF and more women than men [5,6]. At present, pharmacologic treatment for HFpEF includes sodium-glucose cotransporter-2 (SGLT2) inhibitors (Class 1-2a recommendations), mineralocorticoid receptor antagonists (Class 2b recommendations), and angiotensin receptor-neprilysin inhibitors (class 2b recommendations) [1,7]. Despite these options, HFpEF remains difficult to treat because of its marked clinical and pathophysiologic heterogeneity, underscoring the need for additional therapeutic approaches.

In recent years, short-chain fatty acids (SCFAs) have garnered attention as candidate mediators in the pathogenesis and progression of cardiovascular diseases [8]. SCFAs are metabolites produced by the gut microbiota through fermentation of dietary fiber [9]. Chemically, they are carboxylic acids with carbon chain lengths ≤6 (C2–C6), comprising four straight-chain acids (acetate, propionate, butyrate, and valerate) and two branched-chain acids (isobutyrate and isovalerate) [10], with acetate, propionate, and butyrate collectively representing 90–95% of all SCFAs [8,10]. In peripheral circulation, acetate is the most abundant SCFA (20–250 μM), followed by propionate (1.4–13.4 μM) and butyrate (0.5–14.2 μM) [11]. SCFAs act primarily through two major pathways: activation of specific G-protein-coupled receptors (GPCRs) and inhibition of histone deacetylase (HDAC) [12]. These pathways mediate metabolism, immune function, inflammatory responses, and autonomic signaling [13,14]. In addition, SCFAs also enter cells as energy substrates via membrane transporters, thereby directly regulating cellular metabolism and function [15].

Here, we review the roles of SCFAs and their receptors in HFpEF. We summarize the cardiovascular expression of SCFA receptors and discuss how SCFA-related signaling may intersect with key HFpEF mechanisms, including inflammation, metabolic dysregulation, and myocardial stiffness. Given the limited direct evidence in HFpEF, we distinguish disease-specific findings from mechanistic evidence derived from broader cardiovascular and metabolic models and cautiously consider the therapeutic relevance of SCFA-related pathways in HFpEF.

## 2. Methods

We conducted this review using a structured literature search to identify studies examining the role of SCFAs in HFpEF. Electronic databases, including PubMed, Web of Science, and Scopus, were searched from inception to 29 April 2026. We focused on studies published within the past decade, while selectively incorporating earlier seminal works. Using Boolean operators, we implemented a search strategy that combined SCFA-related keywords (“short-chain fatty acids,” “SCFAs,” “acetate,” “propionate,” and “butyrate”) with “gut microbiota,” “heart failure with preserved ejection fraction,” “HFpEF,” and “diastolic dysfunction,” and with related mechanistic and clinical keywords. Original research articles, reviews, and relevant preclinical and clinical studies published in English were considered. We selected studies on the basis of their relevance to SCFA biology, HFpEF pathophysiology, or cardiometabolic and inflammatory mechanisms linked to HFpEF. We also classified evidence into several categories and assessed each study independently against these criteria based on the model system of the original source and this classification. Case reports, editorials, conference abstracts, and non-English articles were excluded.

## 3. SCFA Receptors and SCFAs as HDAC Inhibitors

### 3.1. SCFA Receptors

SCFA receptors are GPCRs located on the plasma membrane and activated by SCFAs for intracellular signaling [16,17]. In humans, five SCFA receptors have been identified (Table 1): GPR41 (free fatty acid receptor [FFAR] 3), GPR43 (FFAR2), GPR109A (hydroxycarboxylic acid receptor [HCAR] 2), olfactory receptor 51E2 (OR51E2; human homolog of mouse Olfr78), and OR51E1 (human homolog of mouse Olfr558) [18]. The available evidence differs substantially by receptor and is strongest for immune, vascular, and metabolic pathways, whereas direct HFpEF-specific evidence remains limited.

GPR43 is detected in immune and intestinal endocrine cells, including monocytes and T cells [13]. It is primarily activated by acetate, propionate, and butyrate, with the highest affinity for acetate [19]. Upon activation, GPR43 couples to Gi/o and Gq proteins, influencing intracellular Ca^2+^ levels and the MAPK pathway [20]. In the context of HFpEF, GPR43 may mechanistically link SCFAs, immune cell activation, adipose inflammation, and endothelial dysfunction, although its disease-specific role has yet to be directly established.

GPR41 has been detected in smooth muscle cells of small resistance vessels, the renal artery, aorta, and iliac artery, as well as in adipocytes, neutrophils, sympathetic ganglia, and kidneys [21]. It is activated particularly by propionate with an EC_50_ of ~10 µM and butyrate with an EC_50_ of ~30 μM. GPR41 activation by butyrate primarily signals through Gi/o proteins, leading to decreased intracellular cAMP and reduced cytosolic Ca^2+^ levels in cardiomyocytes [22]. These effects may be relevant to HFpEF through vascular resistance, sympathetic activation, and cardiomyocyte calcium handling, although their disease-specific role requires direct validation in HFpEF models.

GPR109A, also known as HCAR2, is mainly expressed in epithelium, adipocytes, and monocytes and is activated by butyrate with EC_50_ values of 0.4 to 1.6 mM [23,24]. GPR109A activation signals through Gi/o proteins and downregulates intracellular cAMP levels [25]. Through GPR109A-mediated anti-inflammatory signaling, butyrate may be relevant to myocardial fibrosis and microvascular inflammation, although direct evidence in HFpEF is lacking.

OR51E2 has been identified as one of the most enriched odorant receptor transcripts in human airway smooth muscle cells [26]. In heterologous expression systems, OR51E2 responds selectively to acetate and propionate, with dose-dependent increases in cAMP observed in transfected HEK-293T cells [26]. The estimated EC_50_ for both ligands was approximately 2 mM, about eight-fold higher than the upper reported peripheral (plasma) acetate concentration (250 μM) and far exceeding the reported propionate range (1.4–13.4 μM) [26]. However, local and tissue levels of SCFAs have been reported to be in the range of 0.1–10 mM. Thus, robust activation of OR51E2 requires elevated systemic and/or local SCFA concentrations [21]. Because patients with HFpEF frequently have chronic hypertension, arterial stiffness, and impaired vascular compliance, SCFA-mediated OR51E2 signaling may be relevant to renin release, vascular tone, arterial stiffness, and cardiac afterload; however, this interpretation is based on indirect vascular and renal evidence rather than HFpEF-specific studies. OR51E1 is found primarily in tumor tissues, including gastric, prostate, and colorectal cancers [27,28,29]. Notably, OR51E1 is also expressed in airway smooth muscle cells but at lower levels than OR51E2 [26]. In transfected HEK-293T cells, butyrate induces dose-dependent increases in cAMP and Ca^2+^ levels through Golf protein [30]. OR51E1 may therefore represent an SCFA-responsive metabolic sensing pathway, but its relevance to cardiovascular remodeling and HFpEF remains speculative.

### 3.2. SCFAs as HDAC Inhibitors

SCFAs act as key regulators of epigenetic modulation by inhibiting HDAC. Histone acetylation by acetyltransferases relaxes chromatin, enhancing gene expression, whereas HDAC-mediated deacetylation condenses chromatin, suppressing transcription [31]. Aberrant HDAC activation is implicated in cardiovascular disease, where it contributes to inflammation, fibrosis, endothelial dysfunction, and maladaptive cardiac remodeling [31].

### 3.3. SCFAs as Diverse Molecules with Varied Effects

Importantly, acetate, propionate, and butyrate are not interchangeable (Table 2). Acetate is the most abundant circulating SCFA and preferentially activates GPR43, propionate shows greater activity at GPR41, and butyrate has comparatively prominent HDAC-inhibitory and GPR109A-activating effects [19,23,31]. These differences in abundance, receptor preference, and intracellular metabolism most likely influence dose requirements and biological responses. Therapeutic studies should therefore evaluate each SCFA separately rather than infer compound-specific effects from mixed-SCFA preparations.

## 4. SCFAs and HFpEF

**Figure 1:**
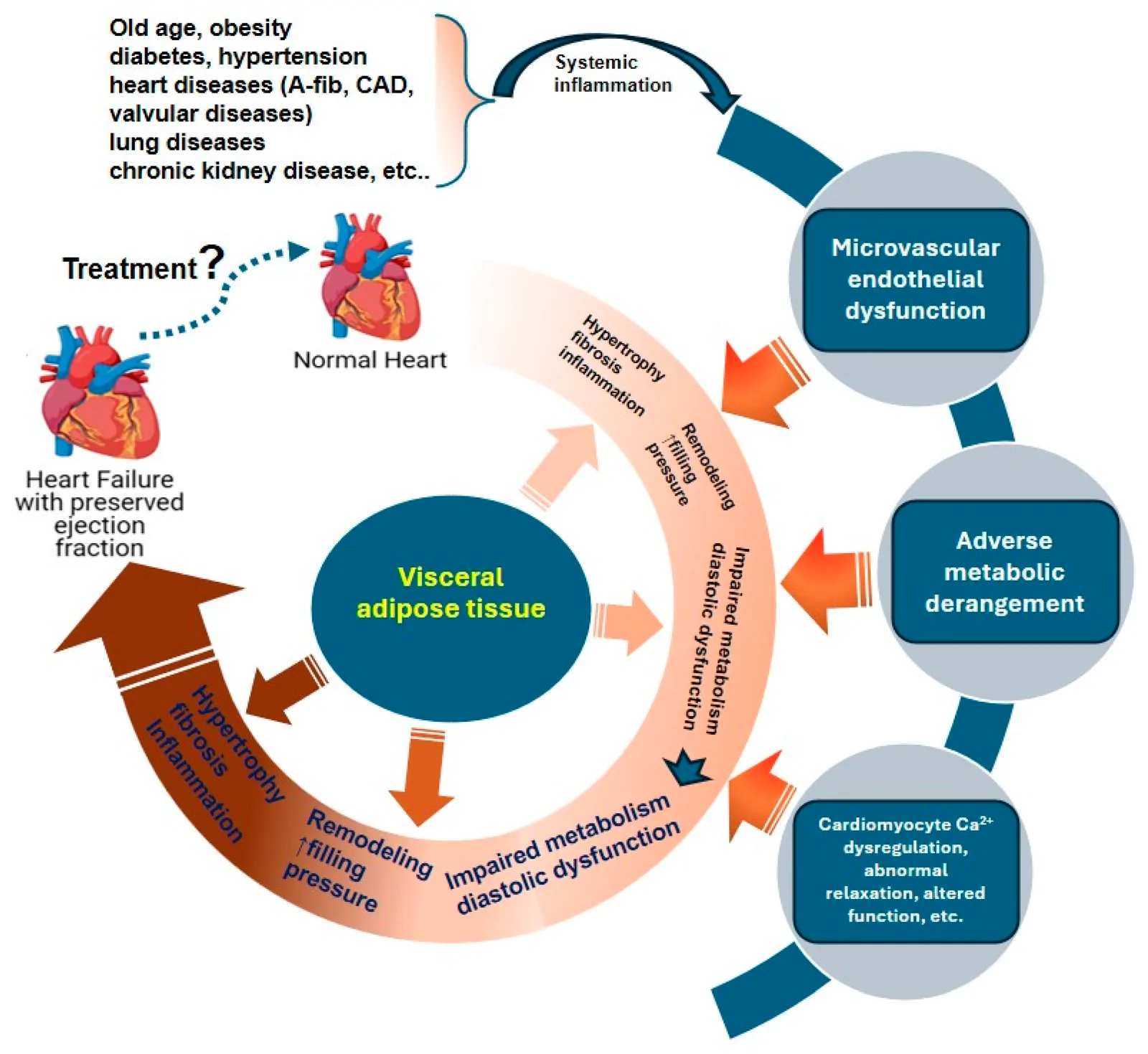
**Overview of mechanism underlying pathogenesis and development of HFpEF.** The presence of disease triggers and risk factors leads to systemic inflammation through activation of inflammatory and immune cells, which secrete inflammatory agents. The increased inflammation causes coronary endothelial cell dysfunction, cardiac metabolic derangement, alterations in cardiac excitation–contraction coupling (Ca^2+^ dysregulation, impaired relaxation, abnormal force development, etc.), and myocardial fibrosis (outer dark blue blocks and large orange arrows). The accumulation of visceral fatty tissue exerts stress on the adipocytes to produce more harmful adipokines, further exacerbating the pathophysiologic processes as a central element (central blue circle and brown circular arrows). As a result, the heart is remodeled both in structure and function, and HFpEF develops and progresses. Currently, therapeutic options for HFpEF remain limited (dotted arrow and question mark). A-fib = atrial fibrillation; CAD = coronary artery disease (Created in BioRender. Yang, X. (2026) https://biorender.com/8woi8tu, accessed on 25 August 2026).

The pathophysiology of HFpEF involves chronic low-grade systemic inflammation driven by multiorgan comorbidities [2,32]. This inflammation contributes to adipokine dysregulation, coronary microvascular endothelial dysfunction, myocardial energetic abnormalities, impaired myocardial relaxation, and interstitial fibrosis (Figure 1) [4,32,33]. These processes are closely linked to reduced nitric oxide (NO)–soluble guanylate cyclase (sGC)–cyclic GMP (cGMP)–protein kinase G (PKG) signaling, increased myocardial stiffness, diastolic dysfunction, and elevated pulmonary pressure—phenotypical features of HFpEF [4,32].

Because SCFAs and their receptors regulate inflammation, endothelial function, and metabolism, they may plausibly intersect with these HFpEF-related pathways (Table 3). However, this mechanistic plausibility should be interpreted cautiously, as direct HFpEF-specific evidence remains limited.

### 4.1. The Potential Role of SCFAs in Modulating Adipokines in HFpEF

A cardinal feature of HFpEF is central obesity, characterized by excessive accumulation of visceral fat, including epicardial adipose tissue, which secretes predominantly harmful adipokines [49]. These adipokines play a central role in the pathophysiology of HFpEF by promoting inflammation, fibrosis, myocardial structural and functional abnormalities, and metabolic dysregulation.

SCFAs have been shown to regulate adipokine secretion and metabolic functions in adipocytes [34]; however, their specific role in HFpEF remains incompletely defined. For instance, studies that used pericardial adipocytes derived from individuals with type 2 diabetes have shown that SCFAs can suppress lipid droplet accumulation and concurrently upregulate the mRNA expression of both adiponectin and leptin [35]. These observations support the hypothesis that SCFAs might influence the obesity–adipokine component of HFpEF, particularly through adiponectin-related metabolic effects and potentially peroxisome proliferator-activated receptor gamma-linked pathways [50]. Nevertheless, the impact of concomitant leptin upregulation, tissue-specific receptor expression, and the inflammatory HFpEF milieu remain uncertain. Therefore, SCFA–adipokine signaling should be viewed as a mechanistic hypothesis that requires direct validation in HFpEF models and patient cohorts.

### 4.2. The Anti-Inflammatory Effects of SCFAs and HFpEF

Chronic low-grade systemic inflammation is a hallmark of patients with HFpEF and one of the earliest drivers of disease progression [51]. Proinflammatory cytokines, such as IL-6, IL-8, monocyte chemoattractant protein 1 (MCP-1), and TNF-α, are significantly elevated in patients with HFpEF [52,53]. These cytokines recruit immune cells, especially macrophages and T lymphocytes, into myocardial tissue, where they activate local inflammatory and fibrotic responses [54]. In HFpEF, adverse remodeling is accompanied by CCR2^+^ proinflammatory (M1-like) macrophage infiltration, as well as elevated inflammatory cytokines and chemokines that promote adverse remodeling [55,56]. Endothelial inflammation and dysfunction exacerbate these effects by reducing NO–cGMP–PKG signaling, thereby increasing myocardial stiffness [57]. Activation of damage signals such as the NF-κB–NLRP3 pathway further intensifies local inflammation and structural remodeling [58], amplifies cytokine production, and promotes monocyte recruitment and macrophage accumulation, driving fibroblast–myofibroblast transformation [59,60]. Consistent with these results, endomyocardial biopsies from patients with HFpEF show increased infiltration of T cells and myeloid inflammatory cells [53].

Direct evidence that SCFAs function as anti-inflammatory agents in HFpEF remains limited. However, some indirect evidence has been reported. For example, SCFA-producing gut microbes were reduced in patients with HFpEF as compared with those in healthy controls [36]. In a mouse model of myocardial infarction, acetate, propionate, and butyrate were decreased in serum and fecal samples, but SCFA supplementation altered myeloid cell composition toward a myocardial protective phenotype [37]. Furthermore, in mouse models of ischemia–reperfusion, effective plasma concentrations of SCFAs not only inhibited the NF-κB pathway in cells expressing GPR41 and GPR43 but also suppressed neutrophil chemotaxis and reduced M1 macrophages [38]. These findings provide a foundation for investigating SCFA-mediated anti-inflammatory mechanisms in HFpEF.

The epigenetic mechanisms of SCFAs may also play an anti-inflammatory role in HFpEF. SCFAs can inhibit HDACs, which have been shown to promote inflammatory gene expression and activate the NLRP3 inflammasome in a human monocyte cell line [61]. Indeed, inhibition of HDAC6 reduced myocardial infiltration of T cells and macrophages, inhibited NF-κB signaling, and suppressed TNF-α, IL-18, and IL-1β levels, thereby alleviating chronic kidney disease-induced myocardial remodeling [39]. Additionally, the HDAC inhibitor butyrate decreased proinflammatory factor expression in macrophages and downregulated NLRP3 activation [62]. These findings suggest that SCFA-mediated HDAC inhibition may counteract inflammatory signaling in HFpEF.

Taken together, these findings suggest that SCFAs may modulate inflammatory pathways relevant to HFpEF through effects on immune cell activity, inflammasome signaling, and epigenetic regulation. However, their direct anti-inflammatory efficacy in HFpEF remains to be established.

### 4.3. Protective Effects of SCFAs on the Endothelium and HFpEF

Microvascular endothelial dysfunction is increasingly recognized as a central link between systemic inflammation and myocardial remodeling in HFpEF [32,63]. Endothelial NO signaling is impaired in HFpEF through multiple mechanisms, including downregulation of endothelial NO synthase (eNOS) activity with lower phosphorylation, eNOS uncoupling induced by oxidative stress, and NO degradation by reactive oxygen species (ROS) [64]. In HFpEF, elevated oxidative stress promotes excessive generation of superoxide (O_2_^•−^) [65]. Superoxide rapidly reacts with NO to form peroxynitrite (ONOO^−^), which oxidizes the essential eNOS cofactor tetrahydrobiopterin and contributes to eNOS uncoupling, further reducing NO synthesis and bioavailability [66]. Depletion of NO reduces cGMP level and PKG signaling [57]. Loss of PKG activity has two major consequences: (1) impaired Ca^2+^ reuptake in cardiomyocytes, which delays relaxation [67], and (2) reduced titin phosphorylation, which increases myocardial passive stiffness [7,68]. Activated endothelial cells upregulate the expression of adhesion molecules such as vascular cell adhesion molecule 1 (VCAM-1) and intercellular adhesion molecule 1 (ICAM-1) to recruit monocytes and promote transendothelial migration [28]. In addition, the depletion of NO, which normally acts as a negative regulator for the NLRP3 inflammasome, amplifies this inflammatory signaling [69].

SCFAs have the potential to restore NO bioavailability and alleviate endothelial dysfunction, both of which can moderate HFpEF pathophysiology [40,47]. In angiotensin II-stimulated aortic endothelial cells, acetate and butyrate enhanced eNOS expression, reduced ROS production from nicotinamide adenine dinucleotide phosphate (NADPH) oxidase and mitochondria, and increased NO levels [40]. The effects of butyrate were mediated, at least in part, through GPR41/43 [40]. Propionate reversed endoplasmic reticular stress-induced reductions in eNOS phosphorylation at Ser1177 and improved NO release in human cardiac microvascular endothelial cells [41]. In animal models, sodium butyrate also improved NO signaling and endothelial function by enhancing eNOS expression and reducing oxidative stress [70,71].

SCFAs also exert anti-inflammatory effects on endothelial cells by modulating cytokine production and adhesion molecule expression [42,43,44]. Acetate inhibited IL-6 and IL-8 expression through GPR43 activation, whereas propionate and butyrate downregulated IL-6, IL-8, VCAM-1, and ICAM-1 expression and reduced monocyte adhesion through GPR41/43 and HDAC inhibition in human umbilical vein endothelial cells [42,43,44]. Though largely cell-based, these findings support a mechanistic link between SCFAs and endothelial inflammatory pathways relevant to HFpEF.

### 4.4. Modulation of Ca^2+^ Handling and Mitochondrial Function by SCFAs and HFpEF

In HFpEF, diastolic Ca^2+^ overload is common owing to impaired Ca^2+^ reuptake by the sarcoplasmic reticulum calcium ATPase (SERCA) 2a and extrusion through the Na^+^/Ca^2+^ exchanger [72] or increased diastolic sarcoplasmic reticular Ca^2+^ leak through hyperphosphorylated ryanodine channels [73,74]. Dysfunction of the SERCA2a and Na^+^/Ca^2+^ exchanger also increases diastolic Ca^2+^ [75]. This Ca^2+^ dysregulation impairs diastolic relaxation [76]. Also in HFpEF, elevated afterload from neurohormonal activation, arterial stiffening, and systemic hypertension increase ATP demand [77]. This excessive energy demand contributes to ROS accumulation and mitochondrial dysfunction [78]. ROS, in turn, activate CaMKII, which sustains inotropy, but its overactivation leads to cardiomyocyte death and hypertrophy [79,80]. Mitochondrial dysfunction limits ATP production and reduces excitation–contraction coupling efficiency, resulting in excitation-energy uncoupling [81,82]. In Zucker fatty and spontaneously hypertensive F1 hybrid (ZSF1) rats, Ca^2+^ overload triggers mitochondrial swelling and complex I dysfunction via mitochondrial Ca^2+^ uniporter-mediated uptake [82].

No studies have systematically assessed the interventional effects of SCFAs on mitochondrial dysfunction in HFpEF. In microglia, acetate has been shown to regulate Ca^2+^ homeostasis, mitochondrial damage, and redox balance by fueling the TCA cycle (through conversion to acetyl-CoA) and supporting mitochondrial oxidative phosphorylation [45]. Moreover, acetate supplementation rescued complex II-related mitochondrial dysfunction and restored mitochondrial quantity and metabolic activity [45]. In another study, propionate improved mitochondrial fission and mitophagy in HT22 cells stimulated with amyloid-β (Aβ) via activation of GPR41 and GPR43 [46]. Further, an SCFA cocktail of acetate, propionate, and butyrate (1 μM) restored mitochondrial membrane potential and respiratory function and reduced ROS generation and Ca^2+^ overload in angiotensin II-stimulated endothelial cells [47]. Collectively, these findings indicate that SCFAs may preserve mitochondrial structure, support ATP synthesis, and regulate Ca^2+^ overload.

## 5. Cellular Targets and Mechanisms Relevant to HFpEF

**Figure 2:**
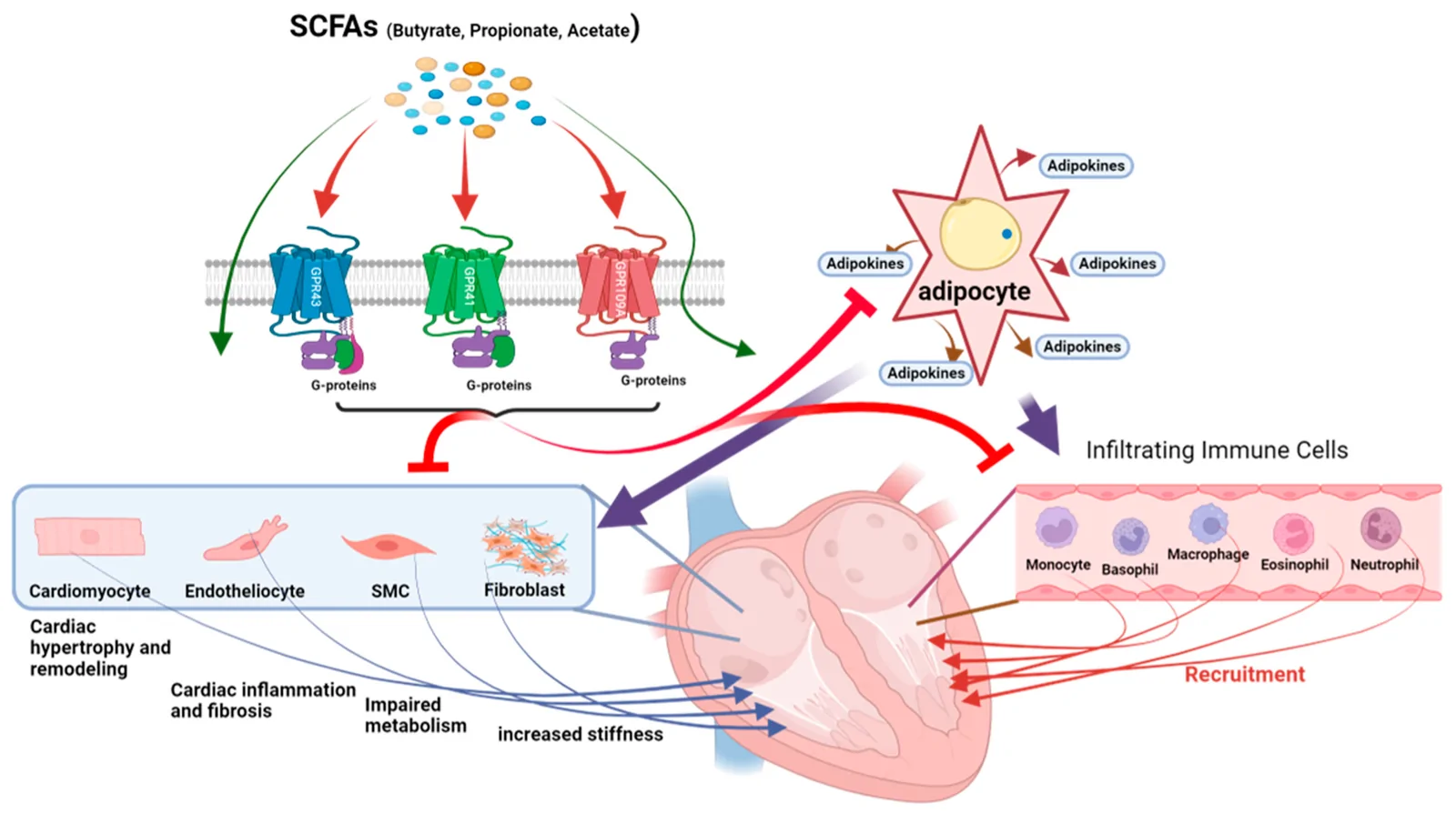
**Potential cellular targets of SCFAs.** SCFAs (butyrate, propionate, and acetate) act on a variety of cell types, such as adipocytes, inflammatory/immune cells, cardiomyocytes, endothelial cells, smooth muscle cells, and fibroblasts, via membrane receptors or direct interaction with targeted proteins. Specifically, the anti-inflammatory, antifibrotic, the immune cell recruitment inhibitory, adipokine modulating, and pro-energetic effects of SCFAs can influence pathways relevant to HFpEF (red T shaped lines), although direct disease-specific evidence remains limited. SMC, smooth muscle cell (Created in BioRender. Yang, X. (2026) https://biorender.com/ikhd5k9, accessed on 25 August 2026).

SCFA receptors and HDAC inhibition by SCFAs can potentially modulate the function of cardiomyocytes, fibroblasts, vascular endothelial cells, and immune cells. In this section, we discuss how the anti-inflammatory, antifibrotic, and pro-energetic effects of SCFAs might prevent HFpEF development or slow its progression (Figure 2).

### 5.1. Cardiomyocytes

Cardiomyocyte dysfunction, particularly impaired diastolic relaxation, is central to the pathophysiology of HFpEF. However, little is known about changes in excitation–contraction coupling processes in HFpEF. One study by Hegemann et al. [83] showed that intracellular Ca^2+^ transient amplitudes are decreased, compensated by increases in myofilament Ca^2+^ sensitivity, whereas Jani et al. [84] showed either decreased or unchanged myofilament Ca^2+^ sensitivity. These metabolic changes might exacerbate the calcium-handling abnormalities described above, further compromising myocardial relaxation and contractile reserve [72].

In animal models, GPR41 and GPR43 are functionally expressed in cardiomyocytes, and their activation by butyrate and acetate can influence intracellular Ca^2+^ handling and myocardial contraction. Recently, we reported that activation of GPR41 by butyrate suppressed cAMP and SERCA activity through a Gαi pathway, reducing Ca^2+^ transients and sarcomere shortening in rat cardiomyocytes [22,48]. Acetate transiently reduced contractility through GPR43 without altering action potentials or Ca^2+^ channel activity, suggesting metabolic modulation [85].

SCFAs might also modulate cardiomyocyte activity via autonomic pathways. Studies have suggested that SCFAs influence sympathetic neurotransmission by reducing norepinephrine release at nerve terminals. This reduction in norepinephrine can indirectly alter myocardial excitability [86].

SCFAs and GPR41/GPR43 may modulate cardiomyocyte function under stress. For example, GPR41 and GPR43 are expressed at low levels under basal conditions in adult human cardiomyocytes but are elevated during myocardial infarction, as revealed by single-cell RNA sequencing data [48,87]. Given the changes in excitation–contraction coupling in HFpEF and the effects of SCFAs and their receptors on cardiomyocytes, cardiomyocyte SCFA signaling remains a testable hypothesis rather than an established HFpEF mechanism.

### 5.2. Cardiac Fibroblasts

Cardiac fibroblasts contribute to interstitial fibrosis and stiffness in HFpEF. SCFAs may directly influence these cells, as transcriptomic analyses revealed that GPR41/43-expressing fibroblast subtypes are responsive to cytokines and transforming growth factor-β (TGF-β) [48,87]. Functional evidence for SCFA-mediated fibroblast modulation comes from a mouse model of granulation tissue formation, in which the metabolite-sensing receptor GPR43 contributed to the effects of low-dose sodium butyrate on angiogenesis and extracellular matrix remodeling [88]. Moreover, GPR43^+^ fibroblasts in nonfibrotic regions of human cardiac tissue were enriched for mitochondrial organization and cytoskeletal stability, suggesting a quiescent or reparative phenotype that may preserve myocardial compliance. Given that myocardial stiffness is a key characteristic of HFpEF, SCFA-mediated modulation of fibroblast activity could be a candidate therapeutic pathway, but it requires direct validation in HFpEF models.

### 5.3. Coronary Microvascular Endothelium and Smooth Muscle Cells

Endothelial dysfunction and vascular rarefaction are cardinal features of HFpEF. These vascular abnormalities may be modulated by SCFA receptors, including GPR41, GPR43, and Olfr78, which are expressed in pericytes, endothelial cells, and smooth muscle cells [14,21,89,90]. A transitional pericyte subtype, characterized by both endothelial and stromal markers (PC3_str), shows high co-expression of GPR43 and GPR41 [48]. Consistent with a protective vascular role, SCFAs reduced vascular rarefaction and interstitial fibrosis in pressure-overload models [91]. These findings suggest that SCFAs may modulate pathways related to microvascular dysfunction, although HFpEF-specific causal evidence remains lacking.

### 5.4. Infiltrating Immune Cells

Chronic inflammation and immune cell infiltration are central to HFpEF pathogenesis, often triggered by comorbidities such as obesity, hypertension, and diabetes. SCFA receptors (GPR41, GPR43, and GPR109A) are robustly expressed in a variety of immune cells within the heart, including monocytes, macrophages, neutrophils, and lymphocytes [48,87]. Activation of GPR43 by SCFAs can increase intracellular Ca^2+^, which is important for immune cell function [92]. Macrophage and neutrophil populations, which contribute to tissue remodeling and inflammation resolution, are particularly enriched with GPR43. Functionally, SCFA signaling via GPR41 and GPR43 modulates cytokine release and immune cell recruitment. In neutrophils, GPR43 activation enhances chemotaxis and calcium flux, whereas in macrophages it inhibits proinflammatory cytokines such as IL-6 and TNF-α. Additionally, SCFAs promote regulatory T cell function and suppress T helper 17 (Th17) responses, suggesting a role in adaptive immunity [92]. These immunomodulatory effects are consistent with the chronic low-grade inflammatory milieu of HFpEF and suggest a plausible link between SCFA–GPCR signaling and immune-mediated myocardial injury. However, this link is hypothesis-generating rather than direct evidence of therapeutic efficacy.

## 6. Preclinical and Clinical Evidence Implicating SCFA Involvement

Investigations linking SCFAs and HFpEF directly are sparse in both preclinical and clinical research (Table 3). Preclinical studies have tested SCFAs only in animal models that did not recapitulate all features of HFpEF. Clinical evidence is limited because SCFA level measurements have been inconsistent and no direct, causal effects of SCFA have been observed in HFpEF.

As shown in Table 4, evidence is classified into several categories: (1) Direct HFpEF evidence includes studies conducted in patients meeting standard HFpEF diagnostic criteria or in validated preclinical HFpEF models. (2) Other disease evidence includes studies that were conducted under conditions that were mechanistically relevant but not in HFpEF-specific models. (3) Mechanistic or cell-based evidence was derived from studies that did not use disease models.

### 6.1. Preclinical Studies in Animal Models

Recent preclinical studies suggest that SCFAs, especially propionate and butyrate, may influence several features of cardiovascular dysfunction that resemble, but do not fully recapitulate, HFpEF. Intravenous sodium propionate reduced left ventricular end-diastolic pressure and perivascular fibrosis in hypertensive rats, despite no significant change in systemic blood pressure [94]. In a mouse model with obesity and insulin resistance, continuous propionate delivery improved cardiomyocyte energy metabolism through GPR41 and AMP-activated protein kinase (AMPK) pathway activation [95]. In hypertensive models, butyrate improved NO availability and attenuated oxidative damage [96]. Under diastolic stress (elevated stiffness of the left ventricle under pressure overload), both acetate and propionate helped preserve ventricular compliance through activation of GPR41/43 receptors; this effect was accompanied by reduced myocardial collagen content [87]. Propionate administration also improved diastolic function, restored ATP production, and stimulated mitochondrial biogenesis in obese mice [98]. Butyrate also improved microvascular function in mice through matrix stabilization and GPR43 signaling [88]. Collectively, these findings support the translational plausibility of SCFA-related pathways, but they do not yet establish SCFA efficacy in validated multimorbidity HFpEF models.

SCFAs mediate many effects through GPCRs, particularly GPR41, GPR43, and GPR109A. Synthetic agonists such as AR420626 (for GPR41) and 4-CMTB (for GPR43) have been developed to enhance potency and receptor selectivity [20]. Activation of GPR41 with AR420626 has been shown to enhance AMPK signaling and mitochondrial respiration in cardiac and skeletal muscle, leading to improved energy efficiency under conditions of pressure overload and metabolic stress [99]. These effects are relevant in HFpEF, where systemic and microvascular inflammation contributes to diastolic dysfunction and myocardial stiffening. GPR109A, activated by both niacin and acipimox, has also been investigated through pharmacologic and genetic approaches. Niacin administration in mice fed a high-fat diet reduced hepatic lipogenesis and intestinal lipid absorption while enhancing thermogenesis in adipose tissue [100]. These receptor-specific tools provide opportunities to target individual SCFA signaling pathways and evaluate their therapeutic potential in HFpEF.

### 6.2. Clinical Evidence

Clinical evidence linking systemic SCFAs to HFpEF remains limited, offering preliminary but associative clinical support for their relevance to HFpEF. In a longitudinal prospective study of 18 patients with newly diagnosed HF, changes in gut microbial composition and fecal SCFAs were associated with clinical evolution over 12 months, although the cohort was not restricted to HFpEF [101]. In a cross-sectional study of patients referred for invasive coronary angiography, fasting plasma propionate concentrations were significantly lower in individuals with confirmed coronary artery disease than in those without [102]. Analyses based on HF type revealed that acetate and propionate levels were lower in patients withHF with reduced ejection fraction (HFrEF), HFpEF, or HF with mildly reduced ejection fraction (HFmrEF) than in healthy controls. Butyrate levels were lower in those with HFmrEF/HFpEF than in healthy controls, but they were higher in patients with HFrEF [93]. These studies support an association between systemic SCFA profiles and HFpEF-relevant diastolic or inflammatory phenotypes, but they do not demonstrate that SCFAs improve diastolic function or clinical outcomes.

Despite growing interest in the role of SCFAs in cardiovascular disease, several important limitations currently restrict the interpretation of human studies in HFpEF. One major challenge is the substantial variability in SCFA measurements across studies, particularly when comparing circulating blood concentrations with fecal/stool concentrations. Stool SCFA levels are often used as a surrogate marker of microbial production; however, fecal concentrations may not accurately reflect systemic SCFA bioavailability because most SCFAs are rapidly absorbed and metabolized in the colon and liver before reaching circulation. Plasma SCFA measurements may better represent biologically active exposure at peripheral tissues, yet circulating SCFAs are highly influenced by diet, fasting status, intestinal absorption, hepatic metabolism, medication use, renal clearance, and sample processing methods. Pre-analytical factors—including collection timing, delay before processing, storage temperature, freeze–thaw cycles, extraction, and derivatization—can alter measured concentrations [103,104]. Analytical platforms such as gas chromatography, LC–MS/MS, and high-resolution MS also differ in sensitivity, calibration, recovery, and limits of quantification [103,104,105]. Studies should therefore report the sample matrix, collection and storage conditions, internal standards, calibration procedures, and quality-control performance. Until these procedures and clinically relevant reference ranges are standardized, between-cohort comparisons and proposed HFpEF thresholds should be interpreted cautiously [105].

A second limitation is the unresolved dilemma regarding the relationship between SCFAs and HFpEF pathogenesis. Although reduced SCFA abundance is associated with HFpEF-relevant features such as inflammation, endothelial dysfunction, hypertension, and metabolic dysregulation, it remains unclear whether impaired SCFA signaling actively contributes to disease development or reflects downstream consequences of established HFpEF. Patients with HFpEF frequently exhibit intestinal edema, venous congestion, impaired gut perfusion, and altered gastrointestinal motility, all of which may disrupt gut microbial composition and reduce SCFA-producing bacterial populations. Consequently, the observed reduction in SCFAs may represent a secondary manifestation of HFpEF-associated gut dysfunction rather than a primary driver of disease progression.

Much of the current evidence linking SCFAs to cardiovascular disease remains associative rather than mechanistic. Most available human studies are cross-sectional and cannot establish temporal relationships or causality. Human HFpEF populations are also highly heterogeneous, with varying comorbidities such as obesity, hypertension, diabetes, renal dysfunction, aging, and systemic inflammation, which complicates efforts to isolate the specific effects of SCFA signaling. Furthermore, many studies infer SCFA activity indirectly through microbiome composition without directly measuring receptor activation, tissue-specific signaling, or downstream cardiovascular effects.

## 7. Knowledge Gaps and Future Directions

As discussed above, preclinical studies have associated SCFAs with anti-inflammatory and immunomodulatory effects, metabolic efficiency improvement, glucotoxicity and lipotoxicity attenuation, blood pressure regulation, and oxidative stress reduction [14,91]. These multidimensional actions make SCFA-related pathways plausible investigational targets in HFpEF. However, these effects have not been systematically tested in established HFpEF models, and no human trial has demonstrated that SCFA-directed intervention improves HFpEF outcomes.

Several preclinical HFpEF models are available, including rodent single-factor models such as aging, hypertension, metabolic obesity, or insulin resistance; multi-hit models such as high-fat diet plus L-NAME and ZSF1 rats; and large-animal or in vitro human systems. These models differ in controllability, comorbidity replication, and clinical translation assessment [106,107,108]. Future studies should use these models to test SCFA-related interventions with predefined endpoints, including diastolic function, microvascular inflammation, NO–sGC–cGMP–PKG signaling, titin phosphorylation, fibrosis, exercise tolerance, and tissue-specific receptor activation.

Clinical evidence is limited, especially in rigorously phenotyped HFpEF cohorts. Most patient data are observational and cross-sectional, providing little insight into temporal relationships and causal inference. There is currently no clear consensus on whether SCFA levels are consistently decreased, increased, or dysregulated in patients with HFpEF, because circulating SCFA levels are highly dynamic and influenced by many variables as described in Section 6.2. Variability may reflect tissue-specific receptor expression and cell-type-specific sensitivity to SCFA signaling, such that local responses may differ from systemic SCFA concentrations. Longitudinal and interventional trials are needed to determine whether modifying SCFA levels through diet, supplementation, targeted microbiome modulation, or pharmacologic receptor activation can improve diastolic function or clinical outcomes in humans. Thus, current studies provide a valuable clinical foundation, but the causal and therapeutic relevance of SCFA pathways in HFpEF remains unproven. Taken together, these gaps show the need to first investigate the effect and underlying mechanisms of SCFAs and their receptors in preclinical HFpEF models and then validate the findings in integrated multimorbidity models that more faithfully recapitulate HFpEF pathophysiology. Building on this work, well-controlled interventional studies in humans will be essential to establish whether SCFAs and their receptors represent clinically actionable therapeutic targets in HFpEF.

## 8. Conclusions

SCFAs and their receptors have multiple biological effects, including adipokine modulation, anti-inflammation, endothelium protection, and regulation of Ca^2+^ handling and mitochondrial function. These effects and associated pathways intersect with HFpEF pathogenesis, indicating that SCFAs can potentially prevent and curb HFpEF development. However, direct human evidence is limited to observational associations between HFpEF and altered SCFA-producing gut bacteria or systemic SCFA profiles. No established therapeutic benefit for SCFAs has been shown. Potential targets at the cellular level include cardiomyocytes, cardiac fibroblasts, cardiac vascular endothelial cells, and immune/inflammatory cells. Indeed, HFpEF-relevant preclinical evidence suggests that SCFAs may influence inflammation, endothelial dysfunction, myocardial fibrosis, and mitochondrial metabolism, but these actions (or interventions) have not been validated in a multimorbidity HFpEF model. Indirect mechanistic findings from non-HFpEF animal models and cell-based systems support GPCR- and HDAC-dependent effects on cardiovascular and metabolic pathways, but these findings cannot be directly extrapolated to patients with HFpEF. Hence, SCFAs and their respective signaling pathways require validation in appropriate preclinical models of HFpEF and prospective human studies before these pathways can be considered as targets for clinical application.

## Figures and Tables

**Table 1:** SCFA receptors, HFpEF-relevant mechanisms, and current levels of evidence.

Receptor	Main Ligands	Relevant Expression/Sites	HFpEF-Relevant Pathway	Evidence Level
GPR43/FFAR2	Acetate, propionate, and butyrate	Immune cells, intestinal endocrine cells, adipocytes, endothelial cells	Immune cell activation, adipose inflammation/adipokine signaling, endothelial inflammation	Mostly cell-based and preclinical evidence; direct receptor-specific HFpEF evidence lacking
GPR41/FFAR3	Propionate and butyrate; acetate with lower potency	Small resistance vessels, adipocytes, neutrophils, sympathetic ganglia, kidney, cardiomyocytes	Vascular resistance, sympathetic regulation, cardiomyocyte Ca^2+^ handling, metabolic signaling	Preclinical vascular and mechanistic cardiomyocyte evidence; HFpEF causality unproven
GPR109A/HCAR2	Butyrate among SCFAs	Epithelium, adipocytes, monocytes/macrophages	Anti-inflammatory signaling, adipose metabolism, fibrosis- and microvascular inflammation-related pathways	Cell-based and preclinical evidence; indirect HFpEF relevance
OR51E2/Olfr78	Acetate and propionate	Airway smooth muscle; vascular and renal renin regulatory sites described for Olfr78	Vascular tone, renin release, arterial stiffness, afterload-related mechanisms	Heterologous and preclinical evidence; indirect HFpEF relevance
OR51E1/Olfr558	Butyrate	Tumor tissues, airway smooth muscle, and other non-cardiac tissues	Metabolic sensing and cAMP/Ca^2+^ signaling	Mainly receptor pharmacology and cell-based evidence; cardiovascular and HFpEF relevance speculative

HFpEF = heart failure with preserved ejection fraction; SCFA = short-chain fatty acid; cAMP = cyclic adenosine monophosphate.

**Table 2:** Features of acetate, propionate, and butyrate.

SCFA	Specificity	Potency (EC_50_)	Biological Effects
Acetate	Main ligand to GPR43, GPR41, OR51E2,	GPR43, 10–500 μM GPR41, 0.4–1.4 mM OR51E2, 2.93 mM	Most abundant; highest water solubility; substrate for energy metabolism; promotes IgA and mucosal immunity; recruits neutrophils and induces IL-22; inhibits T cell migration and activation; anti-inflammatory
Propionate	Main ligand to GPR43, GPR41, OR51E2	GPR43, 250–500 μM GPR41, 6–127 µM OR51E2, 2.16 mM	Stimulates GLP-1 secretion; activates the inflammasome; promotes antimicrobial peptide secretion, anti-inflammation, immunosuppression, epigenetic regulation of metabolism, vascular relaxation
Butyrate	Main ligand to GPR43, GPR41	GPR43, 300–500 µM GPR41, 33–158 µM	Stimulates GLP-1 secretion; acts as energy substrate; promotes mucosal immunity; improves gastrointestinal health; inhibits HDAC; anti-inflammatory

GLP-1 = glucagon-like peptide-1; HDAC = histone deacetylase; SCFA = short-chain fatty acid.

**Table 3:** Effects of SCFAs in experimental models and their potential implications to HFpEF.

Potential Role	Study Model	Main Findings	Implications for HFpEF
Modulating adipokines	Cultured adipocytes [34]	SCFAs dose-dependently inhibited adipokines	Effects depend on SCFA doses and types and on adipokines
	Cultured adipocytes from humans with diabetes [35]	SCFAs stimulated secretion of adiponectin and leptin	SCFAs inhibit lipid accumulation; adiponectin prevents HFpEF; leptin promotes HFpEF
Anti-inflammatory effects	HFpEF patients, MI in mice [36,37]	Reduced numbers of SCFA-producing bacteria; decreased levels of gut SCFAs	In mice, SCFA supplement protected damage by MI via myeloid cell composition modification
	Ischemia–reperfusion mouse models [38]	SCFAs inhibited the NF-κB pathway, suppressed neutrophil chemotaxis, reduced M1 macrophages	NF-κB-NLRP3 inflammatory pathway has been implicated in HFpEF
	CKD-induced myocardial remodeling in mice [39]	Inhibition of HDAC6 reduced myocardial infiltration of T cells and macrophages, inhibited NF-κB signaling	Suppressed inflammation with decreased TNF-α, IL-18, and IL-1β levels alleviates CKD-induced myocardial remodeling
Endothelial protection	Rat aortic endothelial cells (stimulated by angiotensin II) [40]	SCFAs enhanced eNOS expression, reduced ROS production, and increased NO levels	Decreased NO bioavailability is an important contributor to the development of HFpEF
	Human cardiac endothelial cells [41]	Propionate promoted eNOS and improved NO release	Increases in NO can prevent HFpEF pathology
	Human umbilical vein endothelial cells [42,43,44]	Inhibition of cytokine (IL-6, IL-8) and adhesion molecule (VCAM-1, ICAM-1) expression	These inflammatory factors play important roles in the pathogenesis of HFpEF
Regulation of Ca^2+^ handling and mitochondrial function	Microglia [45]	Acetate drove microglial maturation and restored mitochondrial quantity and metabolic activity	Metabolic alterations are components of HFpEF pathogenesis
	HT22 cells [46]	Propionate improved mitochondrial fission and mitophagy	Mitochondrial dysfunction is associated with HFpEF
	Human brain endothelial cells [47]	SCFAs restored mitochondrial membrane potential and respiratory function and reduced ROS and Ca^2+^ overload	Altered mitochondrial metabolism and Ca^2+^ handling are associated with HFpEF pathology
	Rat cardiomyocytes [22,48]	Butyrate inhibited Ca^2+^ transients and contraction and improved relaxation	Diastolic dysfunction is a cardinal feature of HFpEF

HFpEF = heart failure with preserved ejection fraction; CKD = chronic kidney disease; NF-κB = nuclear factor kappa B; NLRP3 = NLR family pyrin domain-containing protein 3; eNOS = endothelial nitric oxide synthase; TNF-α = tumor necrosis factor-α; ICAM-1 = intercellular adhesion molecule 1; MI = myocardial infarction; NO = nitric oxide; ROS = reactive oxygen species; SCFA = short-chain fatty acid; VCAM-1 = vascular cell adhesion molecule 1.

**Table 4:** Evidence hierarchy for SCFA-related findings relevant to HFpEF.

Evidence Category	Representative Findings	HFpEF Interpretation	Examples
HFpEF patients/clinical HF cohorts	HFpEF has been associated with depletion of SCFA-producing gut bacteria; HF phenotype-specific differences in circulating acetate, propionate, and butyrate have also been reported	Directly relevant to patients, but data are observational and not evidence of therapeutic efficacy	[36,93]
Validated HFpEF models	No study identified in this review directly tested SCFA supplementation or receptor-directed treatment in a validated multimorbidity HFpEF model	This remains the principal disease-specific preclinical evidence gap	Section 6
Other cardiovascular/animal models	SCFAs altered inflammation, endothelial function, vascular remodeling, myocardial fibrosis, and diastolic-stress responses in myocardial infarction, hypertension, pressure-overload, and pulmonary-hypertension models	Supports biological plausibility, but these models do not reproduce the full HFpEF phenotype	[37,38,87,94,95,96]
Mechanistic/cell-based evidence	Receptor assays and studies in adipocytes, immune cells, endothelial cells, cardiomyocytes, and fibroblasts demonstrate GPCR-, HDAC-, Ca^2+^-, and metabolism-related effects	Defines candidate mechanisms; clinical and HFpEF-specific effects cannot be inferred directly	[19,22,26,34,40,41,42,43,44,45,46,47,70,71,88,97]

GPCR = G-protein-coupled receptor; HDAC = histone deacetylase; HF = heart failure; HFpEF = heart failure with preserved ejection fraction; SCFA = short-chain fatty acid.

## Data Availability

The original data are included in the manuscript, and further queries can be directed to the corresponding author.
